# Molecular and Morphological Identification of Mealybug Species (Hemiptera: Pseudococcidae) in Brazilian Vineyards

**DOI:** 10.1371/journal.pone.0103267

**Published:** 2014-07-25

**Authors:** Vitor C. Pacheco da Silva, Aline Bertin, Aurélie Blin, Jean-François Germain, Daniel Bernardi, Guylène Rignol, Marcos Botton, Thibaut Malausa

**Affiliations:** 1 Embrapa Uva e Vinho, Bento Gonçalves, Brazil; 2 Escola Superior de Agricultura “Luiz de Queiroz”, Universidade de São Paulo, Piracicaba, Brazil; 3 Institut National de la Recherche Agronomique, Institut Sophia Agrobiotech, UMR INRA / UNSA / CNRS BP 167. 06903, Sophia-Antipolis, France; 4 ANSES, Laboratoire de la Santé des Végétaux, Unité d'entomologie et Plantes Invasives Campus International de Baillarguet, CS 30016, Montferrier-sur-Lez, France; State University of Maringá/Universidade Estadual de Maringá, Brazil

## Abstract

Mealybugs (*Hemiptera: Pseudococcidae*) are pests constraining the international trade of Brazilian table grapes. They damage grapes by transmitting viruses and toxins, causing defoliation, chlorosis, and vigor losses and favoring the development of sooty mold. Difficulties in mealybug identification remain an obstacle to the adequate management of these pests. In this study, our primary aim was to identify the principal mealybug species infesting the major table grape-producing regions in Brazil, by morphological and molecular characterization. Our secondary aim was to develop a rapid identification kit based on species-specific Polymerase Chain Reactions, to facilitate the routine identification of the most common pest species. We surveyed 40 sites infested with mealybugs and identified 17 species: *Dysmicoccus brevipes* (Cockerell), *Dysmicoccus sylvarum* Williams and Granara de Willink, *Dysmicoccus texensis* (Tinsley), *Ferrisia cristinae* Kaydan and Gullan, *Ferrisia meridionalis* Williams, *Ferrisia terani* Williams and Granara de Willink, *Phenacoccus baccharidis* Williams, *Phenacoccus parvus* Morrison, *Phenacoccus solenopsis* Tinsley, *Planococcus citri* (Risso), *Pseudococcus viburni* (Signoret), *Pseudococcus cryptus* Hempel, four taxa closely related each of to *Pseudococcus viburni, Pseudococcus sociabilis* Hambleton, *Pseudococcus maritimus* (Ehrhorn) and *Pseudococcus meridionalis* Prado, and one specimen from the genus *Pseudococcus* Westwood. The PCR method developed effectively identified five mealybug species of economic interest on grape in Brazil: *D. brevipes*, *Pl. citri*, *Ps. viburni*, *Ph. solenopsis* and *Planococcus ficus* (Signoret). Nevertheless, it is not possible to assure that this procedure is reliable for taxa that have not been sampled already and might be very closely related to the target species.

## Introduction

Grapevines cover an area of approximately 82,000 hectares in Brazil, with an annual yield ranging from 1,300,000 to 1,450,000 t [1]. The cultivation of this crop has expanded throughout Brazil, with Rio Grande do Sul state producing the largest amounts, followed by Pernambuco, São Paulo and Paraná. Nationally, 57% of grape production is destined for consumption as table grapes and 43% is used for juice and wine production [1], [2]. Brazilian production levels increased in recent decades, largely due to expansion of the export of table grapes, mostly produced in the northern regions of Brazil, especially the São Francisco Valley, which is responsible for producing 90% of the grapes exported from Brazil [2], [4]. The cities of Petrolina (in Pernambuco) and Juazeiro (in Bahia) are the main grape exporters, and the industry is of utmost importance for the socioeconomic growth of the region [3], [4].

One of the key factors limiting the export of Brazilian grapes is the presence of mealybugs Hemiptera: Pseudococcidae). Mealybugs are small phloem-sucking insects, the nymphs and adult females of which feed by sucking sap from the trunk, roots, leaves, rachis and fruits of grapevines, causing direct and indirect damage, depending on the species and the site used for feeding [5]–[9]. The mere presence of these pests in the harvested and shipped fruits is a major cause of quarantine rejections [8], [10]. Moreover, dense populations of mealybugs may decrease plant vigor, cause defoliation and introduce toxic substances into the leaves, triggering chlorosis. Furthermore, the chief damage inflicted by these pests results from their transmission of viruses affecting final product quality and vineyard longevity [11]–[15]. In addition to all these other types of damage, mealybugs reduce the marketability of table grapes by excreting honeydew, which promotes the development of sooty mold on fruits.

Daane *et al*. [9] reviewed the complex of vineyard mealybugs worldwide, five species of which are important in Brazil: the citrus mealybug *Planococcus citri* (Risso), the pineapple mealybug *Dysmicoccus brevipes* (Cockerell), the vine mealybug *Pseudococcus viburni* (Signoret), the grape mealybug *Pseudococcus maritimus* (Ehrhorn) and the passionvine mealybug *Planococcus minor* Maskell. These mealybugs are morphologically very similar and are therefore difficult to tell apart, particularly for non specialists. Current methods for distinguishing between mealybug species are based on observations of the morphological characteristics of adult female specimens under the microscope. This method is particularly time-consuming, impracticable for males and nymphs, and may be inconclusive, particularly for very closely related species [15]–[17]. This is particularly problematic because difficulties in identification may jeopardize the use of control and management methods specific to certain target species, which are currently favored over the use of broad-spectrum pesticides. For example, biological control methods based on the release of natural enemies (e.g. hymenopteran parasitoids) or pheromone trapping systems are mostly species-specific and require correct pest identification [18]. Moreover, different mealybug species cause greatly different degrees of damage and not all species are considered to be quarantine species, depending on the market to which fruits are exported.

Given the difficulties involved in identifying mealybugs morphologically and the importance of correct identification, the association of DNA sequencing with morphological identification is particularly useful, making identification quicker, cheaper and more reliable. Several genomic regions have successfully been used for the identification of mealybugs and other scale insects. These regions include 28S-D2 and internal transcribed spacer 2 (ITS2) in the nuclear DNA, the mitochondrial cytochrome oxidase subunit I (COI) gene, and the leuA-16S region located in the DNA of the primary endosymbionts of most Pseudococcidae, *Tremblaya princeps* [17]–[26]. Furthermore, the DNA sequencing data obtained can be used to develop species-specific Polymerase Chain Reactions (PCR), making it possible to identify species molecularly, on the basis of the size of the sequence amplified [18], [27]–[29].

We used a combination of DNA sequencing at five loci and morphological characterization to survey the mealybug species infesting 40 Brazilian vineyards located in three major grape-producing regions, based on the methods described by Malausa *et al*. [24] and Abd-Rabou *et al*. [18]. We then designed a multiplex PCR method for rapid identification of the five mealybug species most commonly found or considered to be a major threat to Brazilian vineyards.

## Materials and Methods

### Sample collections

Forty-eight samples, each containing one to 20 mealybug individuals, were collected from Brazilian table grapes vineyards (from vines or other plants in the immediate vicinity of vines within the vineyards) during 2008 and 2009. Mealybugs were collected from the aerial parts of the plants or from the roots. Samples were collected from 40 sites in the states of Rio Grande do Sul, Pernambuco and Paraná, in order to carry out collections in major grape-producing regions of Brazil. The number of sites is smaller than the number of samples, because mealybugs collected from different plants within the same vineyard were considered to constitute different samples. Specimens were stored in ethanol (95%) at −20°C for identification and molecular analyses. GPS coordinates, host plants and the mealybug identifications are present in Table 1.

**Table 1:** List of populations sampled: Population code, geographic origin and host origin of the samples.

Population code	City	GPS coordinates	Host plant	Collection date	Identification
1	Caxias do Sul	29°09.964' S, 51°06.596′ W	*Vitis vinifera*	29/04/2009	*Pseudococcus viburni*
2	Caxias do Sul	29°08.023′ S, 51°06.140′ W	*Vitis vinifera*	06/05/2009	*Pseudococcus viburni*
3	Caxias do Sul	29°16.093′ S, 51°01.906′ W	*Vitis vinifera*	29/04/2009	*Pseudococcus* nr. *maritimus*
4	Caxias do Sul	29°15.567′ S, 51°09.980′ W	*Vitis vinifera*	07/05/2009	*Dysmicoccus texensis*
5	Caxias do Sul	29°15.376′ S, 51°10.684′ W	*Vitis vinifera*	07/05/2009	*Pseudococcus viburni*
6	Caxias do Sul	29°09.964′ S, 51°06.596′ W	*Rumex* sp.	29/04/2009	*Pseudococcus viburni, Pseudococcus* nr. *viburni, Dysmicoccus brevipes*
7	Caxias do Sul	29°13.135′ S, 51°14.832′ W	*Rumex* sp.	21/05/2009	*Dysmicoccus brevipes*
8	Caxias do Sul	29°13.459′ S, 51°08.461′ W	*Rumex* sp.	06/05/2009	*Dysmicoccus brevipes*
9	Caxias do Sul	29°14.787′ S, 51°16.474′ W	*Rumex* sp.	21/05/2009	*Dysmicoccus brevipes*
10	Caxias do Sul	29°13.826′ S, 51°01.012′ W	*Rumex* sp.	29/04/2009	*Dysmicoccus sylvarum*
11	Caxias do Sul	29°16.093′ S, 51°01.906′ W	*Rumex* sp.	29/04/2009	*Dysmicoccus sylvarum*
12	Caxias do Sul	29°13.288′ S, 51°01.249′ W	*Vitis vinifera*	29/04/2009	*Pseudococcus viburni*
13	Caxias do Sul	29°15.818′ S, 51°11.224′ W	*Vitis vinifera*	05/06/2009	*Pseudococcus viburni*
14	Bento Gonçalves	29°09.853′ S, 51°31.777′ W	*Vitis vinifera*	19/02/2009	*Dysmicoccus brevipes*
15	Bento Gonçalves	29°09.853′ S, 51°31.777′ W	*Sonchus oleraceus*	07/01/2009	*Pseudococcus viburni*
16	Caxias do Sul	29°15.871′ S, 51°11.074′ W	*Vitis vinifera*	09/10/2008	*Pseudococcus* sp.
17	Caxias do Sul	29°10.375′ S, 51°05.511′ W	*Vitis vinifera*	29/04/2009	*Pseudococcus viburni*
18	Caxias do Sul	29°13.955′ S, 51°16.914′ W	*Vitis vinifera*	21/05/2009	*Pseudococcus viburni*
19	Marialva	23°30.015' S, 51°49.628' W	*Vitis vinifera*	22/07/2009	*Planococcus citri*
20	Marialva	23°30.856' S, 51°47.535' W	*Vitis vinifera*	21/07/2009	*Pseudococcus* nr. *sociabilis, Planococcus citri*
21	Sarandi	23°21.520′ S, 51°48.561' W	*Bidens pilosa*	22/07/2009	*Planococcus citri*
22	Mandaguari	23°31.784' S, 51°41.638' W	*Vitis vinifera*	22/07/2009	*Planococcus citri, Phenacoccus parvus*
23	Pinto Bandeira	29°06.358′ S, 51°28.987′ W	*Vitis vinifera*	28/04/2009	*Dysmicoccus brevipes*
24	Sarandi	23°26.775' S, 51°48.293' W	*Bidens pilosa*	22/07/2009	*Phenacoccus parvus*
25	Petrolina	09°14.313' S, 40°27.475 W	*Vitis vinifera*	10/04/2008	*Planococcus citri, Phenacoccus solenopsis, Ferrisia meridionalis, Dysmicoccus brevipes*
26	Marialva	23°30.149' S, 51°44.847' W	*Vitis vinifera*	22/07/2009	*Planococcus citri*
27	Marialva	23°27.817' S, 51°47.297' W	*Vitis vinifera*	22/07/2009	*Planococcus citri*
28	Jandaia do Sul	23°38.919' S, 51°37.881' W	*Vitis vinifera*	21/07/2009	*Planococcus citri*
29	Sarandi	23°21.401' S, 51°48.476' W	*Vitis vinifera*	22/07/2009	*Planococcus citri*, *Pseudococcus* nr. *sociabilis, Ferrisia cristinae*
30	Caxias do Sul	29°07.100′ S, 51°12.513′ W	*Vitis vinifera*	28/05/2009	*Ferrisia terani*
31	Caxias do Sul	29°05.473′ S, 51°13.007′ W	*Vitis vinifera*	28/05/2009	*Pseudococcus viburni*
32	Marialva	23°30.558' S, 51°48.963' W	*Vitis vinifera*	22/07/2009	*Planococcus citri, Pseudococcus cryptus*
33	Caxias do Sul	29°07.562′ S, 51°13.695′ W	*Vitis vinifera*	20/05/2009	*Ferrisia meridionalis, Ferrisia terani, Phenacoccus baccharidis*
34	Marialva	23°31.164' S, 51°49.372' W	*Vitis vinifera*	22/07/2009	*Planococcus citri, Ferrisia cristinae*
35	Petrolina	09°15.793' S, 40°36.648' W	*Vitis vinifera*	07/10/2009	*Planococcus citri*
36	Sarandi	23°21.520′ S, 51°48.561' W	*Vitis vinifera*	22/07/2009	*Planococcus citri*
37	Caxias do Sul	29°14.787′ S, 51°16.474′ W	*Vitis vinifera*	21/05/2009	*Phenacoccus baccharidis*
38	Pinto Bandeira	29°07.236' S, 51°27.002' W	*Rumex* sp.	23/04/2009	*Dysmicoccus brevipes*
39	Caxias do Sul	29°08.014′ S, 51°13.969′ W	*Vitis vinifera*	20/05/2009	*Pseudococcus viburni*
40	Petrolina	09°14.404' S, 40°27.881' W	*Vitis vinifera*	08/10/2009	*Planococcus citri*
41	Marialva	23°30.246' S, 51°49.323' W	*Vitis vinifera*	22/07/2009	*Planococcus citri*
42	Petrolina	09°20.733' S, 40°36.767' W	*Malva* sp.	07/10/2009	*Phenacoccus solenopsis*
43	Sarandi	23°21.401' S, 51°48.476' W	*Sonchus oleraceus*	22/07/2009	*Pseudococcus* nr. *meridionalis*
44	Petrolina	09°14.313' S, 40°27.475' W	*Vitis vinifera*	04/06/2008	*Dysmicoccus brevipes*
45	Petrolina	09°23.136' S, 40°38.130' W	Species not identified	21/01/2009	*Phenacoccus solenopsis*
46	Marialva	23°30.015' S, 51°49.628' W	Species not identified	22/07/2009	*Phenacoccus parvus*
47	Caxias do Sul	29°16.045′ S, 51°02.166′ W	*Vitis vinifera*	29/04/2009	*Pseudococcus viburni*
48	Marialva	23°30.496' S, 51°49.048' W	*Vitis vinifera*	22/07/2009	*Planococcus citri*

All samplings were conducted in private areas, except the sampling done in the Bento Gonçalves city (Table 1, population codes 14 and 15) which were carried out at the research center of Embrapa Grape and Wine (Brazilian Agricultural Research Corporation; responsible person for the permit: Dr. Marcos Botton, marcos.botton@embrapa.br). No specific permission was required for the sampling in other areas. Most collected species are well-known agricultural pests that cause damage to crops and are widely distributed. None of them is an endangered species.

### DNA extraction, amplification and sequencing

When possible, we analyzed five mealybug individuals from each of the samples collected. In total, we extracted DNA from 215 mealybugs, with the DNeasy Blood and Tissue Kit (QIAGEN, Valencia, CA). We ensured that voucher specimens were available for morphological identification, by not crushing the specimens before extraction. Instead, we used the non-destructive method described by Malausa *et al*. [23].

We aimed to amplify and sequence five DNA loci known to be informative for species identification and providing sufficient data for the subsequent design of species-specific PCR primers for the identification kit (see next section): two slightly overlapping parts of the cytochrome oxidase subunit I (COI) gene, 28S-D2, internal transcribed spacer 2 (ITS2), and the leuA-16S region of the DNA of the symbiont *Tremblaya princeps*. Except for the first region of the COI gene (the LCO-HCO region used in most international DNA barcoding projects), for which we used an updated version of the primers [17], we followed the protocol described by Malausa *et al*. [23]. The primers used (Forward, Reverse) were 5′AGAGAGAGTTCAAGAGTACGTG3′ and 5′TTGGTCCGTGTTTCAAGACGGG3′ for 28S-D2, 5′CTCGTGACCAAAGAGTCCTG3′ and 5′TGCTTAAGTTCAGCGGGTAG3′ for ITS2; 5′YAATATAATRATTACWWTWCATGC3′ and 5′TTTWCCATTTAAWGTTATTATTC3′ for the first region of COI hereafter referred to as “LCO”; 5′CAACATTTATTTTGATTTTTTGG3′ and 5′GCWACWACRTAATAKGTATCATG3′ for the second region of COI hereafter referred to as “C1”; and 5′GTATCTAGAGGNATHCAYCARGAYGGNG3′ and 5′GCCGTMCGACTWGCATGTG3′ for leuA-16S. The annealing temperature for these primer pairs was 58°C for 28S-D2 and ITS2, 48°C for LCO, 56°C for C1 and leuA-16S. The PCR conditions were provided in a previous study [23], and are kept updated at http://bpi.sophia.inra.fr/dnabarcoding/.

PCR was performed with a 23 µl reaction mixture and 2 µl of diluted DNA (1–20 ng of DNA matrix). The reagent concentrations were 1×Phusion HF buffer (Phusion High-Fidelity DNA polymerase 530 [Thermo Fisher Scientific, Vantaa, Finland]), 0.01 U/µl Phusion enzyme, 200 µM dNTPs and 0.5 µM of each primer.

For bidirectional sequencing, all PCR products were sent to Genoscreen (Lille, France) or to the French National Genoscope (Paris, France) for capillary electrophoresis on ABI automatic sequencers (Applied Biosystems, Foster City, CA, USA). Consensus sequences were generated and checked with Seqscape v2.7 (ABI). Alignments were edited manually with Bioedit version 7.01 [30].

Sequences were compared by direct alignment, and any specimen sequence with a different nucleotide present at one or more positions was considered to constitute a different haplotype. We also used Haplotype Detector software (http://www2.sophia.inra.fr/urih/sophia_mart_fr/genotyping_tools.php) to distinguish between and sort the various haplotypes automatically. The sequences analyzed were deposited in GenBank for future access and use (Table 2).

**Table 2:** GenBank accession number, Blast Hits, corresponding taxon, % similarity and coverage between the Brazilian mealybug sequences and sequences from the NCBI GenBank database.

Haplotype (GenBank accession #)	Identification (DNA + morphology)	Best GenBank BLAST hit	Corresponding taxon	% similarity	Coverage (bp)
28S-01 (KJ530578)	*Dysmicoccus brevipes*	GU134658.1	*Dysmicoccus brevipes*	100%	321
28S-02 (KJ530579)	*Planococcus citri*	JF714181.1	*Planococcus citri*	100%	310
28S-03 (KJ530580)	*Ferrisia meridionalis*	AY179461.1	*Ferrisia gilli*	99%	314
28S-04 (KJ530581)	*Ferrisia terani*	AY179469.1	*Ferrisia terani*	99%	309
28S-05 (KJ530582)	*Ferrisia cristinae*	AY179464.1	*Ferrisia cristinae*	100%	308
28S-06 (KJ530583)	*Pseudococcus viburni*	GU134653.1	*Pseudococcus viburni*	100%	319
28S-07 (KJ530584)	*Pseudococcus* nr. *viburni*	GU134653.1	*Pseudococcus viburni*	99%	319
28S-08 (KJ530585)	*Pseudococcus viburni*	GU134652.1	*Pseudococcus viburni*	100%	319
28S-09 (KJ530586)	*Pseudococcus cryptus*	GU134654.1	*Pseudococcus comstocki*	96%	321
28S-10 (KJ530587)	*Pseudococcus* nr. *sociabilis*	AY427312.1	*Pseudococcus maritimus*	98%	315
28S-11 (KJ530588)	*Pseudococcus* nr. *sociabilis*	GU134653.1	*Pseudococcus viburni*	94%	323
28S-12 (KJ530589)	*Phenacoccus solenopsis*	JQ085532.1	*Phenacoccus solenopsis*	100%	317
28S-13 (KJ530590)	*Phenacoccus parvus*	GU134663.1	*Phenacoccus parvus*	100%	317
28S-14 (KJ530591)	*Phenacoccus baccharidis*	AY427337.1	*Phenacoccus madeirensis*	89%	321
28S-15 (KJ530592)	*Dysmicoccus texensis*	AY427323.1	*Dysmicoccus neobrevipes*	99%	318
28S-16 (KF804137)	*Pseudococcus* nr. *maritimus*	GU134653.1	*Pseudococcus viburni*	96%	320
28S-17 (KJ530593)	*Dysmicoccus sylvarum*	AY427359 1	*Dysmicoccus* sp.	94%	323
28S-18 (KJ530594)	*Pseudococcus* sp.	GU134655.1	*Pseudococcus* nr. *maritimus*	97%	317
28S-19(KJ530595)	*Pseudococcus* nr. *meridionalis*	GU134655.1	*Pseudococcus* nr. *maritimus*	100%	315
16S-01 (KJ530566)	*Pseudococcus viburni*	JF714174.1	*Pseudococcus viburni*	100%	1003
16S-02 (KJ530567)	*Pseudococcus* nr. *maritimus*	GU134644.1	*Pseudococcus* nr. *maritimus*	97%	1017
16S-03 (KJ530568)	*Dysmicoccus texensis*	GU134650.1	*Dysmicoccus brevipes*	97%	1007
16S-04 (KJ530569)	*Pseudococcus* nr. *viburni*	JF714174.1	*Pseudococcus viburni*	99%	1003
16S-05 (KJ530570)	*Dysmicoccus brevipes*	GU134650.1	*Dysmicoccus brevipes*	100%	994
16S-06 (KJ530571)	*Dysmicoccus sylvarum*	GU134644.1	*Pseudococcus* nr. *maritimus*	96%	1016
16S-07 (KJ530572)	*Planococcus citri*	JF714171.1	*Planococcus citri*	100%	1003
16S-08 (KJ530573)	*Pseudococcus* nr. *sociabilis*	GU134644.1	*Pseudococcus* nr. *maritimus*	98%	1014
16S-09 (KJ530574)	*Ferrisia terani*	JF714173.1	*Dysmicoccus boninsis*	93%	1023
16S-10 (KJ530575)	*Pseudococcus cryptus*	GU134648.1	*Pseudococcus comstocki*	97%	1017
16S-11 (KJ530576)	*Ferrisia meridionalis*	JF714173.1	*Dysmicoccus boninsis*	92%	1023
16S-12 (KJ530577)	*Ferrisia cristinae*	JF714173.1	*Dysmicoccus boninsis*	93%	1022
LCO-20 + C1-05 (KJ530600)	*Dysmicoccus brevipes*	JQ085558.1	*Dysmicoccus brevipes*	99%	760
LCO-23 + C1-06 (KJ530601)	*Dysmicoccus brevipes*	JQ085558.1	*Dysmicoccus brevipes*	99%	760
LCO-26 + C1-19 (KJ530602)	*Dysmicoccus sylvarum*	JQ085558.1	*Dysmicoccus brevipes*	89%	760
LCO-27 + C1-10 (KJ530603)	*Dysmicoccus sylvarum*	JQ085558.1	*Dysmicoccus brevipes*	89%	760
C1-21 (KJ530604)	*Ferrisia meridionalis*	AY179445.1	*Ferrisia pitcairnia*	94%	384
LCO-22 + C1-22 (KJ530605)	*Ferrisia terani*	JQ085554.1	*Ferrisia virgata*	92%	760
LCO-21 + C1-22 (KJ530606)	*Ferrisia terani*	JQ085554.1	*Ferrisia virgata*	92%	760
LCO-14 + C1-13 (KJ530607)	*Ferrisia cristinae*	JQ085554.1	*Ferrisia virgata*	92%	760
C1-12 (KJ530608)	*Ferrisia cristinae*	AY179448.1	*Ferrisia cristinae*	99%	385
LCO-24 + C1-24 (KJ530609)	*Phenacoccus parvus*	GU134711.1	*Phenacoccus parvus*	97%	740
C1-23 (KJ530610)	*Phenacoccus solenopsis*	AB858432.1	*Phenacoccus solenopsis*	100%	362
LCO-01 + C1-03 (KJ530611)	*Planococcus citri*	JQ085542.1	*Planococcus citri*	99%	760
LCO-02 + C1-04 (KJ530612)	*Planococcus citri*	JQ085542.1	*Planococcus citri*	99%	760
LCO-03 + C1-01 (KJ530613)	*Planococcus citri*	JQ085543.1	*Planococcus citri*	99%	760
LCO-04 + C1-01 (KJ530614)	*Planococcus citri*	JQ085543.1	*Planococcus citri*	100%	760
LCO-04 + C1-02 (KJ530615)	*Planococcus citri*	JQ085543.1	*Planococcus citri*	100%	760
LCO-05 + C1-01 (KJ530616)	*Planococcus citri*	JQ085543.1	*Planococcus citri*	99%	760
LCO-19 + C1-14 (KJ530617)	*Pseudococcus cryptus*	JQ085562.1	*Pseudococcus comstocki*	94%	760
LCO-07 + C1-11 (KJ530618)	*Pseudococcus viburni*	JQ085549.1	*Pseudococcus viburni*	99%	760
LCO-08 + C1-10 (KJ530619)	*Pseudococcus viburni*	JQ085549.1	*Pseudococcus viburni*	99%	760
LCO-09 + C1-08 (KJ530620)	*Pseudococcus viburni*	JQ085549.1	*Pseudococcus viburni*	99%	760
LCO-10 + C1-09 (KJ530621)	*Pseudococcus viburni*	JQ085549.1	*Pseudococcus viburni*	98%	760
LCO-11 + C1-09 (KJ530622)	*Pseudococcus viburni*	JQ085549.1	*Pseudococcus viburni*	98%	760
LCO-12 (KJ530623)	*Pseudococcus viburni*	JQ085549.1	*Pseudococcus viburni*	98%	760
LCO-06 + C1-07 (KJ530624)	*Pseudococcus viburni*	JQ085549.1	*Pseudococcus viburni*	100%	760
LCO-15 + C1-28 (KJ530625)	*Pseudococcus* nr. *viburni*	JQ085549.1	*Pseudococcus viburni*	93%	760
C1-16 (KJ530626)	*Pseudococcus* nr. *sociabilis*	JF714166.1	*Pseudococcus viburni*	92%	431
LCO-13 + C1-15 (KJ530627)	*Pseudococcus* nr. *sociabilis*	JQ085554.1	*Ferrisia virgata*	93%	760
LCO-17 + C1-18 (KJ530628)	*Dysmicoccus texensis*	JQ085558.1	*Dysmicoccus brevipes*	93%	760
C1-25 (KJ530629)	*Pseudococcus* nr. *meridionalis*	GU134683.1	*Pseudococcus* nr. *maritimus*	99%	368
LCO-16 + C1-29 (KJ530630)	*Pseudococcus* sp.	JQ085549.1	*Pseudococcus viburni*	92%	760
LCO-28 + C1-26 (KJ530631)	*Phenacoccus baccharidis*	HM474264.1	*Phenacoccus solani*	89%	649
LCO-25 + C1-27 (KJ530632)	*Pheanacoccus baccharidis*	JQ085562.1	*Pseudococcus comstocki*	90%	760
LCO-18 + C1-17 (KJ530633)	*Pseudococcus* nr. *maritimus*	JQ085562.1	*Pseudococcus comstocki*	91%	760
					
ITS2-01 (KF804140)	*Dysmicoccus sylvarum*	JX228132.1	*Dysmicoccus neobrevipes*	71%	716
ITS2-02 (KF804141)	*Dysmicoccus texensis*	JX228133.1	*Dysmicoccus neobrevipes*	90%	704
ITS2-03 (KJ530596)	*Ferrisia cristinae*	JQ085571.1	*Ferrisia virgata*	73%	957
ITS2-04 (KJ530597)	*Ferrisia cristinae*	JQ085571.1	*Ferrisia virgata*	72%	959
ITS2-05 (KF804144)	*Dysmicoccus brevipes*	GU134673.1	*Dysmicoccus brevipes*	100%	723
ITS2-06 (KF804154)	*Ferrisia meridionalis*	JQ085571.1	*Ferrisia virgata*	71%	1032
ITS2-07 (KF804146)	*Ferrisia terani*	JQ085571.1	*Ferrisia virgata*	72%	958
ITS2-08 (KF819646)	*Pseudococcus* nr. *meridionalis*	JF776370.1	*Pseudococcus meridionalis*	99%	774
ITS2-09 (KF819647)	*Pseudococcus* nr. *sociabilis*	JF758861.1	*Pseudococcus maritimus*	85%	741
ITS2-10 (KF819648)	*Pseudococcus* nr. *maritimus*	JN983134.1	*Pseudococcus cribata*	79%	338
ITS2-11 (KJ530598)	*Phenacoccus parvus*	JQ085570.1	*Phenacoccus parvus*	99%	582
ITS2-12 (KF819650)	*Phenacoccus solenopsis*	JQ085569.1	*Phenacoccus solenopsis*	98%	551
ITS2-13 (KF819651)	*Phenacoccus solenopsis*	JQ085569.1	*Phenacoccus solenopsis*	99%	652
ITS2-14 (KF819652)	*Planococcus citri*	HM628576.1	*Planococcus citri*	99%	737
ITS2-15 (KF819653)	*Pseudococcus viburni*	AF006820.1	*Pseudococcus affinis*	100%	754
ITS2-16 (KJ530599)	*Pseudococcus viburni*	AF006820.1	*Pseudococcus affinis*	99%	756
ITS2-17 (KF819655)	*Pseudococcus* nr. *viburni*	AF006820.1	*Pseudococcus affinis*	89%	730
ITS2-18 (KF819656)	*Phenacoccus baccharidis*	JF714191.1	*Phenacoccus peruvianus*	89%	54
ITS2-19 (KF819657)	*Phenacoccus baccharidis*	JX228135.1	*Phenacoccus solenopsis*	93%	95
ITS2-20 (KF819658)	*Pseudococcus* sp.	GU134667.1	*Pseudococcus viburni*	79%	579

For the sequences of LCO and C1, overlapping and covering around 750 bp of Cytochrome Oxidase Subunit I, the contig sequence was used for the Blast study (when both sequences were available).

For rough species delimitation, we used the online version of ABGD - Automatic Barcode Gap Discovery (http://wwwabi.snv.jussieu.fr/public/abgd/), a tool that detects gaps in the sequence barcodes and limits the differences between groups, which are smaller between specimens from the same species and larger for specimens from different species [31]. We used a prior maximal distance P = 1.67 and a Jukes-Cantor MinSlope distance = 1.000000.

We carried out BLAST searches of the GenBank database from NCBI (http://www.ncbi.nlm.gov/BLAST) to identify similarities between our sequence dataset and sequences already published in the GenBank online database. For 28S, COI and 16S, we used the MEGABLAST method (for highly similar sequences), whereas we used the BLASTn method for ITS2.

**Figure 1:**
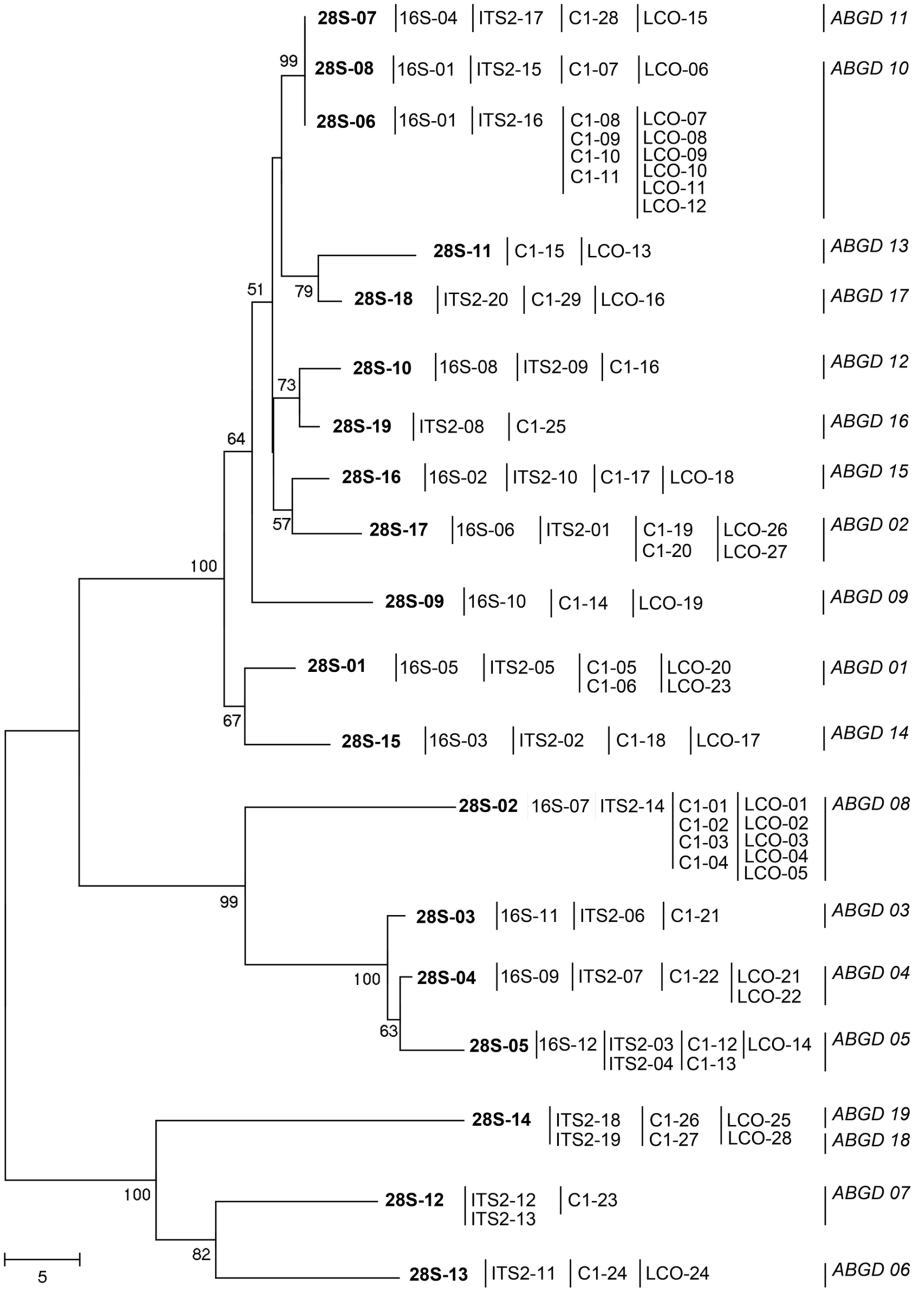
Neighbor-joining tree calculated from the number of differences between 28S haplotypes. Bootstrap values (1,000 replications) are displayed. The 28S alignment used to compute the tree (Figure S1 in File S1) differs from the alignment of raw sequences, because regions including numerous insertions/ deletions were removed to achieve a satisfactory alignment. The 16S, ITS2, C1 and LCO haplotypes of specimens displaying each of the 28S haplotypes are given after the 28S haplotype code.

We generated a neighbor-joining tree based on the number of nucleotide differences between the multilocus haplotypes, with Mega4 [32], to provide a visual representation of the data (this tree was not generated to provide phylogenetic information) (Figure 1).

### Morphological examination

A few specimens displaying each combination of haplotypes (each multilocus haplotype) were slide-mounted for morphological examination and kept as voucher specimens. The slide-mounting process (using the cuticles after the DNA extraction) and morphological examination were carried out by JF Germain, using the protocol previously described by Malausa *et al*. [23]. Morphological identifications were based on four taxonomic keys [33]–[36]. The slide-mounted specimens were deposited in the national collection of ANSES, *Laboratoire de la Santé des Végétaux* (Montferrier-sur-Lez, France) under the code numbers presented in Table 3, and the other specimens were stored in 96% ethanol. DNA extracts were stored at INRA Sophia Antipolis, 400 route des Chappes, Sophia Antipolis, France.

**Table 3:** Summary of mealybug species identified, populations sampled (see Table 1) and different haplotypes obtained for each genetic marker. Different haplotypes obtained for the same species are shown in bold.

Multilocus haplotype	Population sampled	Identification	Slide-mounted specimens	28S	16S	LCO	C1	ITS2
MLH 01	06, 07, 08, 09, 14, 23, 38	*Dysmicoccus brevipes*	902316, 902317, 902318, 902319, 902320, 902321, 902322, 902335, 1002170, 1200840, 1200905, 1200906	28S-01	16S-05	**LCO-20**	**C1-05**	ITS2-05
MLH 02	25, 44	*Dysmicoccus brevipes*	1200841, 1200842, 1200843	28S-01	16S-05	**LCO-23**	**C1-06**	ITS2-05
MLH 03	10	*Dysmicoccus sylvarum*	902323, 902324, 902325	28S-17	16S-06	**LCO-26**	**C1-19**	ITS2-01
MLH 04	11	*Dysmicoccus sylvarum*	902326, 902327, 902328, 902329, 902330	28S-17	16S-06	**LCO-27**	**C1-20**	ITS2-01
MLH 05	25, 33	*Ferrisia meridionalis*	1200858, 1200859, 1200860, 1200861	28S-03	16S-11		C1-21	ITS2-06
MLH 06	30	*Ferrisia terani*	1200863	28S-04	16S-09	**LCO-22**	C1-22	ITS2-07
MLH 07	33	*Ferrisia terani*	1200864	28S-04	16S-09	**LCO-21**	C1-22	ITS2-07
MLH 08	34	*Ferrisia cristinae*	1200867	28S-05	16S-12	LCO-14	**C1-13**	**ITS2-04**
MLH 09	29	*Ferrisia cristinae*	1200865, 1200866	28S-05	-	-	**C1-12**	**ITS2-03**
MLH 10	22, 24, 46	*Phenacoccus parvus*	1200885, 1200886, 1200887	28S-13	-	LCO-24	C1-24	ITS2-11
MLH 11	25, 42	*Phenacoccus solenopsis*	1200879, 1200880, 1200882	28S-12	-	-	C1-23	**ITS2-12**
MLH 12	25, 42, 45	*Phenacoccus solenopsis*	1200881, 1200883, 1200884	28S-12	-	-	C1-23	**ITS2-13**
MLH 13	19, 20, 21, 22, 25, 28, 35, 36, 40	*Planococcus citri*	1101834, 1101836, 1101837	28S-02	16S-07	**LCO-01**	**C1-03**	ITS2-14
MLH 14	29, 41, 48	*Planococcus citri*	1200848, 1200855, 1200856, 1200857	28S-02	16S-07	**LCO-02**	**C1-04**	ITS2-14
MLH 15	40	*Planococcus citri*	1200852, 1200853, 1200854	28S-02	16S-07	**LCO-03**	**C1-01**	ITS2-14
MLH 16	26, 27, 32, 34	*Planococcus citri*	1200844, 1200845, 1200846, 1200850, 1200851	28S-02	16S-07	**LCO-04**	**C1-01**	ITS2-14
MLH 17	29	*Planococcus citri*	1200849	28S-02	16S-07	**LCO-04**	**C1-02**	ITS2-14
MLH 18	27	*Planococcus citri*	1200847, 1200894	28S-02	16S-07	**LCO-05**	**C1-01**	ITS2-14
MLH 19	32	*Pseudococcus cryptus*	1200874, 1200875, 1200876, 1200877	28S-09	16S-10	LCO-19	C1-14	
MLH 20	12	*Pseudococcus viburni*	902331, 1101681	**28S-06**	16S-01	LCO-07	**C1-11**	**ITS2-16**
MLH 21	13	*Pseudococcus viburni*	1101682	**28S-06**	16S-01	LCO-08	**C1-10**	**ITS2-16**
MLH 22	47	*Pseudococcus viburni*	1200871	**28S-06**		LCO-09	**C1-08**	
MLH 23	13	*Pseudococcus viburni*	902332	**28S-06**	16S-01	LCO-10	**C1-09**	**ITS2-16**
MLH 24	05, 47	*Pseudococcus viburni*	1200870, 902309, 1200908, 902310, 902311	**28S-06**	16S-01	LCO-11	**C1-09**	**ITS2-16**
MLH 25	18	*Pseudococcus viburni*	1101832	**28S-06**	-	LCO-12	**-**	**ITS2-16**
MLH 26	01, 02, 06, 15, 17, 18, 31, 39	*Pseudococcus viburni*	902302, 902303, 902305, 902306, 902314, 1200862, 1200873	**28S-08**	16S-01	LCO-06	**C1-07**	**ITS2-15**
MLH 27	6	*Pseudococcus* nr. *viburni*	902313, 902315	28S-07	16S-04	LCO-15	C1-28	ITS2-17
MLH 28	20, 29	*Pseudococcus* nr. *sociabilis*	1101839, 1200878	**28S-10**	16S-08	-	**C1-16**	ITS2-09
MLH 29	20	*Pseudococcus* nr. *sociabilis*	1101838	**28S-11**	-	LCO-13	**C1-15**	-
MLH 30	4	*Dysmicoccus texensis*	902308	28S-15	16S-03	LCO-17	C1-18	ITS2-02
MLH 31	43	*Pseudococcus* nr. *meridionalis*	1200890, 1200891, 1200892, 1200893	28S-19	-	-	C1-25	ITS2-08
MLH 32	16	*Pseudococcus* sp.	1101830	28S-18	-	LCO-16	C1-29	ITS2-20
MLH 33	33	*Phenacoccus baccharidis*	1200888	28S-14	-	**LCO-28**	**C1-26**	**ITS2-19**
MLH 34	37	*Phenacoccus baccharidis*	1200889	28S-14	-	**LCO-25**	**C1-27**	**ITS2-18**
MLH 35	3	*Pseudococcus* nr. *maritimus*	902307	28S-16	16S-02	LCO-18	C1-17	ITS2-10

### Design of species-specific primers for the molecular identification kit

We used SP-Designer [37] software to design species-specific primers, using the list of haplotypes (for each locus studied, separately) as input data.

Briefly, SP Designer designs PCR primers that (i) should hybridize to only a set of sequences targeted by the user (e.g. all the sequences displayed by the individuals of one target species), and (ii) allow the amplification of a DNA fragment of the desired size.

We designed PCR primers hybridizing specifically to the sequences of five species. The first four species targeted were those found in this study to be the most common in Brazil (see results): *D. brevipes, Ps. viburni, Pl. citri* and *Ph. solenopsis.* The fifth species, *Planococcus ficus* (Signoret), constitutes a major threat to vineyards worldwide, and was therefore also selected so that the identification kit would rapidly detect its occurrence in cases of new invasions.

### Species-specific amplification assay

We checked the specificity of the designed primer pairs for the targeted species, by testing each primer pair in PCRs with one or two samples from among the entire set of species surveyed in Brazil (see results). A multiplex PCR was then designed, with one primer pair per species. The primers were selected by testing various primer combinations in PCR with the various Brazilian species surveyed and checking the clarity of the results obtained by electrophoresis of the PCR products. All PCRs were performed with the Multiplex PCR Master Mix (QIAGEN), with a reaction mixture consisting of 1 x PCR Master Mix and primers (0.4 µM each), made up to a final volume of 10 µl with ultrapure water. The PCR conditions were as follows: initial denaturation for 15 min at 95°C; followed by 35 cycles of denaturation for 30 s at 94°C, annealing for 90 s at 62°C, extension for 90 s at 72°C; and a final extension for 30 min at 72°C. PCR products were separated on a QIAxcel advanced system (QIAGEN), and analyzed with QIAxcel ScreenGel Software (QIAGEN).

## Results

### DNA and morphological characterization

We obtained 779 consensus DNA sequences from Brazilian mealybugs, making it possible to analyze 195 specimens. 178 sequences were obtained for 28S (19 different haplotypes), 123 sequences for 16S (12 different haplotypes), 135 sequences for LCO (28 different haplotypes), 183 sequences for C1 (29 different haplotypes) and 160 sequences for ITS2 (20 different haplotypes) (Table S1 in File S1). We observed 35 different multilocus haplotypes in total, corresponding to 19 different taxonomic groups, as defined by ABGD output (Figure 1).

All specimens of ABGD group 1 were morphologically identified as the root mealybug *Dysmicoccus brevipes* (Cockerell). Two multilocus haplotypes (MLH 01 and MLH 02) were observed, with variation observed only for COI. We obtained BLAST hits with Genbank sequences corresponding to *D. brevipes*, with sequences similarities of between 99 and 100%.

The second group (MLH 03 and MLH 04) was morphologically identified as *Dysmicoccus sylvarum* Williams and Granara de Willink. Genetic variation was observed only for COI. As this was the first time that DNA from *D. sylvarum* had been sequenced, no BLAST hits for this species were obtained with GenBank.

Groups 3, 4 and 5 correspond to three different species from the genus *Ferrisia* Fullaway. Group 3 was identified morphologically as *Ferrisia meridionalis* Williams, and contained only one multilocus haplotype (MLH 05). A strong BLAST hit (99%) was obtained for the 28S-03 sequence and a sequence assigned to *Ferrisia gilli* Gullan by Gullan *et al*. [38]. For the other markers, no clear BLAST hit (with similarity >95%) was observed. Group 4 (MLH 06 and MLH 07) was morphologically identified as *Ferrisia terani* Williams and Granara de Willink. BLAST results with C1-22 and 28S-04 revealed hits with GenBank sequences assigned to *F. terani*, with sequence similarities of 97% and 99%, respectively. Group 5 (MLH 08 and MLH 09) was morphologically identified as *Ferrisia* sp. The BLAST hits with the highest degree of sequence similarity corresponded to GenBank sequences assigned to *F. cristinae* Kaydan and Gullan (Table 2) according to the last taxonomic revision of the genus [35].

Group 6 (MLH 10) were morphologically identified as *Phenacoccus parvus* Morrison. BLAST hits revealed similarities of between 97% and 100% with *Ph. parvus* sequences identified in previous studies (Table 2).

Group 7 (MLH 11 and MLH 12) was identified morphologically as *Phenacoccus solenopsis* Tinsley. Again, BLAST hits were associated with high levels of sequence similarity (98% to 100%).

Group 8 (MLH 13 to MLH 18) was morphologically identified as the citrus mealybug, *Planococcus citri* (Risso). In total, 65 specimens were identified as *Pl. citri* in this work, making this species the most frequently observed in Brazilian vineyards. Genetic variation was particularly common in this group, with six multilocus haplotypes observed and differences detected for four of the five markers used. BLAST results revealed hits with very similar sequences (99 to 100%) to a sample previously identified as *Pl. citri*.

Group 9 (MLH 19) was morphologically identified as *Pseudococcus cryptus* Hempel. The BLAST study revealed one hit (99% similarity) with haplotype C1-14 and a *Ps. cryptus* sequence from Genbank. For LCO-19, 28S-09 and 16S-10, we observed hits with similarities of 94, 96 and 97%, respectively, with *Pseudococcus comstocki* Kuwana from Malausa *et al*. [23] and Abd-Rabou *et al.* [17].

Group 10 (MLH 20 to MLH 26) was morphologically identified as the obscure mealybug, *Pseudococcus viburni* (Signoret), intragroup variation was observed, with two 28S haplotypes (28S-6 and 28S-8) and different haplotypes at LCO, C1 and ITS2 associated with each 28S haplotype. All the haplotypes observed displayed similarity to *Ps. viburni* sequences previously published in GenBank (Table 2).

Group 11 (MLH 27) was morphologically identified as *Pseudococcus* near *viburni*. BLAST hits revealed similarities of 99% for 28S-07 and 16S-04 with *Ps. viburni* sequences identified in previous studies (Table 2).

Groups 12 and 13 (MLH 28 and MLH 29) were identified morphologically as *Pseudococcus* near *sociabilis* Hambleton. BLAST hits corresponding to 98% similarity were obtained between the 28S-10 and 16S-08 sequences and GenBank sequences assigned to *Pseudococcus maritimus* (Ehrhorn) by Gullan *et al*. [38] and Malausa *et al*. [23]. However, relatively high levels of divergence (e.g. 5% (14/278) between 28S-10 and 28S-11 and 10% (36/362) between C1-15 and C1-16) were observed between groups 12 and 13, which may thus correspond to two different species that could not be clearly identified as *Ps. sociabilis*.

Group 14 was morphologically identified as *Dysmicoccus texensis* (Tinsley). Only one specimen was sampled (displaying the MLH 30 haplotype). This was the first time that DNA from *D. texensis* has been sequenced. The BLAST hits with the highest scores were obtained for the 16S-03 and 28S-15 loci, with the species *D. brevipes* (97%) and *Dysmicoccus neobrevipes* Beardsley (99%), respectively.

Group 15 (MLH 35) was identified as closely related to the grape mealybug *Ps. maritimus*. For this group, no BLAST hit with a high percentage similarity was found.

Group 16 (MLH 31) was morphologically identified as *Pseudococcus* near *meridionalis* Prado. BLAST hits showed 100% similarity between the 28S-19 sequence and that of *Ps*. near *maritimus* from the study by Malausa *et al*. [23] and 99% similarity between the ITS2-08 sequence and that of *Pseudococcus meridionalis* Prado described by Correa *et al.* [22]. However, not all the characters listed in the description of *Ps. meridionalis* [22] were visible in the specimens collected in this study.

The specimens of group 17 (MLH 32) could not be identified to species level, but were found to belong to genus *Pseudococcus* Westwood. The highest degree of similarity (97%) was that between the 28S-18 sequence and a sequence from *Ps.* near *maritimus* described by Malausa *et al*. [23].

Groups 18 (MLH 33) and 19 (MLH 34) were both identified morphologically as *Phenacoccus baccharidis* Williams. As this species had not been sequenced before, no BLAST hit with a high percentage similarity was detected. The highest similarity observed was 91% between the C1-27 haplotype and *Phenacoccus pergandei* Cockerell, as described by Yokogawa and Yahara [39] (Table 2).

**Figure 2:**
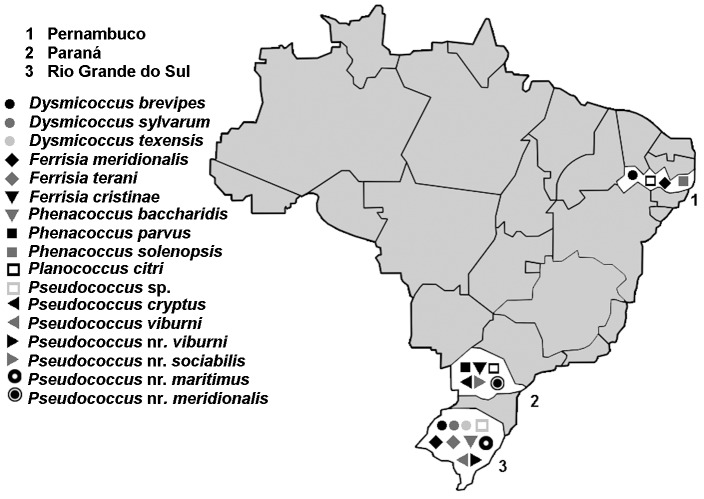
Distribution of mealybug species in vineyards in the Paraná, Pernambuco and Rio Grande do Sul states (Brazil).

The results summarizing the distribution of the various taxa identified in the three grapevine-producing regions of Brazil are provided in Figure 2.

### Identification kit

**Figure 3:**
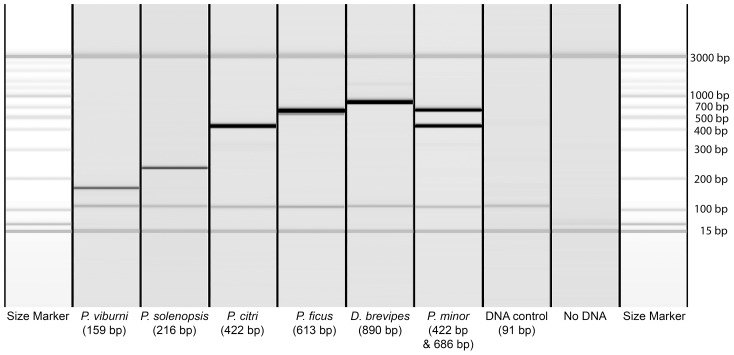
Electrophoresis profile obtained with the Qiaxcel Advanced System for each PCR product from the identification kit. Each lane corresponds to one sample, and each band to a DNA fragment. The first and last lanes contain a molecular weight ladder extending from 50

The seven multiplexed primer pairs (Table 4) yielded PCR products of a particular size for each species when used with the target DNA: 150 bp for *Ps. viburni*, 220 bp for *Ph. solenopsis*, 420 bp for *Pl. citri*, 590 bp for *Pl. ficus*, 890 bp for *D. brevipes,* and a positive control band for the presence of Pseudococcidae DNA at 90 bp (Figure 3). The reaction was found to be specific for the target species, whether that species was obtained from the Brazilian samples studied here or from other samples collected in France and Egypt (including the species surveyed by Abd-Rabou *et al*., [17]).

**Table 4:** Primers used for PCR amplification.

Mealybug species	DNA region	Fragment size (bp)	Forward primer (5′ – 3′)	Reverse primer (5′ – 3′)
*Pseudococcus viburni*	COI	159	CAGCAACTATAATTATTGCTATTCCAACTAG	TAGAAAGAATAATTCCTGTRAAACCACC
*Phenacoccus solenopsis*	28S-D2	216	TTTCTTCGTCGGACGTTTG	AAAGCCGATCTACGCTTCAG
*Planococcus citri* and *Planococcus minor*	ITS2	422	GATGGTTGCGTTCTCGCG	GACGGCGGTAACGTTAAGC
*Planococcus ficus*	ITS2	613	CATGCCAGAGTGATGCGA	AGTACGCTTATAACGCGAATTGA
*Planococcus minor*	COI	686	CCGGTTGAACACTTTATCCC	AGTTAATCCTCCTAATGTAAATATAATGATG
*Dysmicoccus brevipes*	leuA-16S	890	TAGGGAAGCTTTCCGGTACC	TCCAGTTTACGACGTAGGCG
Control for the presence of DNA	18S	91	CAACTGTCGACGGTAGGTTCG	CCGTTTCTCAGGCTCCCTCT

## Discussion

### DNA analyses

Overall, genetic differences between haplotypes clearly separated several taxa, consistent with the results obtained with the ABGD method [31]. We obtained satisfactory congruence between the groups defined by the ABGD method and the morphological identifications of the specimens. In most cases, the occurrence of several multilocus haplotypes assigned to a same ABGD group probably resulted from intraspecific variation, such as observed at the cytochrome oxidase I locus (LCO and C1 regions) for species collected from several sites, such as *Pl. citri*, *D. brevipes* or *D. sylvarum*.

However, in at least three cases, we observed a discrepancy between the results of the different techniques. First, specimens from two ABGD groups were identified morphologically as *Ph*. *baccharidis*. In this case, the occurrence of a unique haplotype at the *28S* locus, the low level of genetic divergence and the morphological homogeneity of the specimens were not consistent with the occurrence of two different species, as proposed by the ABGD method.

Conversely, the two ABGD groups that were both identified as *Ps*. near *sociabilis* morphologically are more likely to correspond to two different unidentified species, both morphologically similar to *Ps. sociabilis*, given the sequence divergence observed for all the loci sequenced for both species (Figure 1). This work is currently inconclusive as concerns the delineation of the various *Pseudococcus* species (*Ps*. near *viburni*, *Ps*. near *maritimus*, *Ps*. near *meridionalis*, *Ps.* near *sociabilis*), but further progress will require a complete re-examination of this species because the morphological characteristics displayed by the collected samples differed from the descriptions of all species by at least a few characteristics. These differences may actually correspond to intraspecific variation, but the collection of samples from various sites and their comparison with the type specimens of each species would be required to improve identification. Unfortunately, only one or a few adults of these species were collected in this study.

The third case is that of the ABGD group identified morphologically as *Ps. viburni*. This group is actually composed of two subgroups, with small fixed differences at all markers other than LeuA-16S, the most strongly conserved marker used in this study. The first subgroup (consisting of all multilocus haplotypes containing 28S-6) displayed remarkable genetic diversity at LCO and C1, whereas the second subgroup had a unique multilocus haplotype 28S-08, 16S-01, LCO-06, C1-07 and ITS2-15. The second subgroup actually includes haplotypes also found in France, Italy, Spain, and Chile [17], [23], [25], [26], whereas the haplotypes from the first subgroup had previously been observed only in southern Brazil (as in this study) by Malausa *et al*. [23]. Hence, the first subgroup may therefore correspond to a species closely related to *Ps. viburni*, endemic to Brazil. Regardless of the actual status of this taxon, the genetic diversity observed in Southern Brazil within populations morphologically identified as *Ps. viburni* supports the hypothesis of Charles [40] about the species being of Neotropical origin.

### Geographic distribution

*Pl. citri, D. brevipes* and *F. meridionalis* were each found in more than one region. *Pl. citri* was sampled from 18 different populations in Paraná and Pernambuco. Interestingly, this species was not observed in Rio Grande do Sul. In Brazil, *Pl. citri* is also a major pest of *Coffea* sp. [41]–[44], occasionally occurs in *Citrus* sp. [45] and has been found in the wine grapes in Rio Grande do Sul [9], [46]. In the State of Paraná, a high level of intraspecific variation was observed, with five different multilocus haplotypes (MLH13, MLH14, MLH16, MLH17 and MLH18), whereas only two multilocus haplotypes were found in Pernambuco (MLH13 and MLH15). The root mealybug, *D. brevipes*, was observed in Rio Grande do Sul and Pernambuco with different multilocus haplotypes: MLH01 in Rio Grande do Sul, and MLH02 in Pernambuco. This species is an important pest of pineapple *Ananas comosus* (L.) Merrill, and, according to the scale insect database ScaleNet, it has previously been observed in several Brazilian states [9], [47]. *F. meridionalis* was observed in Pernambuco and Rio Grande do Sul, the same multilocus haplotype being identified in both regions. This is the first record of this species in Brazil, but it has previously been found in Argentina, Chile, Paraguay and Uruguay [35].

We identified 13 specimens from Pernambuco as *Ph. solenopsis*. This species was recently observed in Brazil on tomato *Solanum lycopersicum* Linnaeus, and then on plants from the Amaranthaceae and Caricaceae families in Espírito Santo State [48], [49].

*Ps. viburni, D. sylvarum, D. texensis*, *F. terani*, *Ph. baccharidis* and *Ps.* near *maritimus* were observed only in Rio Grande do Sul. *Ps. viburni* was very frequently found in this region, with 34 specimens identified at 12 sites. This species has also previously been reported to be present in Minas Gerais, Rio de Janeiro, Rio Grande do Sul, São Paulo and Espírito Santo State [47], [48]. *D. sylvarum* is a species first described in 1992 in Costa Rica [36], subsequently being described for the first time in Brazil in 2006, also sampled in weeds and found in the same region of Rio Grande do Sul State [50]. In this study, we found *D. sylvarum* on vineyard weeds of the genus *Rumex* L. *D. texensis* and *Ph. baccharidis* had already been observed in Brazil, in the states of Minas Gerais and Rio Grande do Sul [34], [36]. For *F. terani,* this is the first record of its presence in Brazil.

In samples from Paraná, the species *F. cristinae*, *Ps. cryptus* and *Ps.* near *sociabilis* were sampled from grape plants. Nine specimens were identified as *Ph. parvus*, from three populations collected from grape plants and weeds. *Ps.* near *meridionalis* was sampled from vineyard weeds of the species *Sonchus oleraceus* Linnaeus. *Ps. meridionalis* is a recently described species first reported in Chilean vineyards (Correa *et al.,* [22]).

The species *Pseudococcus longispinus* (Targioni-Tozzetti), *Ps. maritimus*, and *Pl. minor*, which are major vineyard mealybugs worldwide [9], [51] were not observed in this study.

### Identification kit

The species-specific multiplex PCR successfully detected the four most abundant mealybug species in Brazilian vineyards and the principal threat, *Pl. ficus*, which is already present in Uruguay, close to southern regions of Brazil. The kit was extensively tested on specimens of 29 species found in Brazil, France and Egypt. This kit was found to be suitable for rapid and cost-efficient surveys in Brazilian vineyards. Moreover, the use of positive control PCR primers detecting Pseudococcidae DNA makes it possible to distinguish between an absence of signal due to poor DNA extraction and a lack of signal due to the specimen belonging to a non-target species. However, it is not possible to guarantee that this method is 100% reliable for use with DNA from taxa that have not yet been sampled but are very closely related to the target species.

## Conclusions

The taxonomic identifications obtained in the DNA analyses were entirely consistent with the morphological characterization, allowing the clear identification of 17 species from Brazilian vineyards. *Pl. citri, D. brevipes* and *Ps. viburni* were the most frequently collected species. *F. terani* and *F. meridionalis* were reported for the first time in Brazil. The data and samples obtained from this survey were used to design an identification kit based on five multiplexed species-specific PCRs. This multiplex PCR proved useful for the rapid and cost-efficient identification of *Ps. viburni, Pl. citri, D. brevipes, Ph. solenopsis* and *Pl. ficus.*

## Supporting Information

File S1 **This file includes Figure S1 and Table S1.** Figure S1. 28S sequence alignment used to calculate the Neighbor joining tree of Figure 1. Regions of the alignment with insertions / deletions are removed. Table S1. Summary of molecular and morphological identification of mealybug populations sampled in Brazilian vineyards.
(DOCX)
