# Supplementary material for: Molecular and Morphological Identification of Mealybug Species (Hemiptera: Pseudococcidae) in Brazilian Vineyards
Source: PLoS One. 2014 Jul 25;9(7):e103267. doi: 10.1371/journal.pone.0103267 (PMC4111580; doi:10.1371/journal.pone.0103267)
Supplement: File S1 — This file includes Figure S1 and Table S1. Figure S1. 28S sequence alignment used to calculate the Neighbor joining tree of Figure 1. Regions of the alignment with insertions / deletions are removed. Table S1. Summary of molecular and morphological identification of mealybug populations sampled in Brazilian vineyards. (DOCX) [file pone.0103267.s001.docx]

**SUPPORTING ONLINE MATERIAL**

Table S1.

| **Population Sampled** | **Final identification** | **DNA voucher** | **Slide mounted specimens** | **Haplotypes multilocus** | **28S** | **16S** | **LCO** | **C1** | **ITS2** |
| --- | --- | --- | --- | --- | --- | --- | --- | --- | --- |
| 6 | *Dysmicoccus brevipes* | 1233 | 0902316 | MLH01 | 28S-01 | 16S-05 | LCO-20 | C1-05 | ITS2-05 |
| 6 | *Dysmicoccus brevipes* | 1234 | 0902317 | MLH01 | 28S-01 | 16S-05 | LCO-20 | C1-05 | ITS2-05 |
| 7 | *Dysmicoccus brevipes* | 1235 | 0902318 | MLH01 | 28S-01 | 16S-05 | LCO-20 | C1-05 | ITS2-05 |
| 7 | *Dysmicoccus brevipes* | 1236 | 0902319 | MLH01 | 28S-01 | 16S-05 | LCO-20 | C1-05 | ITS2-05 |
| 7 | *Dysmicoccus brevipes* | 1237 | 1200905 | MLH01 | 28S-01 | 16S-05 | LCO-20 | C1-05 | ITS2-05 |
| 8 | *Dysmicoccus brevipes* | 1239 |  | MLH01 | 28S-01 | 16S-05 | LCO-20 | C1-05 | ITS2-05 |
| 8 | *Dysmicoccus brevipes* | 1240 |  | MLH01 | 28S-01 | 16S-05 | LCO-20 | C1-05 | ITS2-05 |
| 8 | *Dysmicoccus brevipes* | 1241 | 0902320 | MLH01 | 28S-01 | 16S-05 | LCO-20 | C1-05 | ITS2-05 |
| 8 | *Dysmicoccus brevipes* | 1242 | 1200906 | MLH01 | 28S-01 | 16S-05 | LCO-20 | C1-05 | ITS2-05 |
| 8 | *Dysmicoccus brevipes* | 1243 |  | MLH01 | 28S-01 | 16S-05 | LCO-20 | C1-05 | ITS2-05 |
| 9 | *Dysmicoccus brevipes* | 1244 | 0902321 | MLH01 | 28S-01 | 16S-05 | LCO-20 | C1-05 | ITS2-05 |
| 9 | *Dysmicoccus brevipes* | 1245 |  | MLH01 | 28S-01 | 16S-05 | LCO-20 | C1-05 | ITS2-05 |
| 9 | *Dysmicoccus brevipes* | 1246 | 0902322 | MLH01 | 28S-01 | 16S-05 | LCO-20 | C1-05 | ITS2-05 |
| 9 | *Dysmicoccus brevipes* | 1247 |  | MLH01 | 28S-01 | 16S-05 | LCO-20 | C1-05 | ITS2-05 |
| 9 | *Dysmicoccus brevipes* | 1248 |  | MLH01 |  | 16S-05 | LCO-20 | C1-05 |  |
| 14 | *Dysmicoccus brevipes* | 1265 | 0902333 |  |  | 16S-05 |  | C1-05 |  |
| 14 | *Dysmicoccus brevipes* | 1266 |  |  | 28S-01 | 16S-05 |  | C1-05 |  |
| 14 | *Dysmicoccus brevipes* | 1267 |  |  | 28S-01 | 16S-05 |  |  | ITS2-05 |
| 14 | *Dysmicoccus brevipes* | 1268 | 0902335 | MLH01 | 28S-01 | 16S-05 | LCO-20 | C1-05 | ITS2-05 |
| 14 | *Dysmicoccus brevipes* | 1269 | 1002170 |  | 28S-01 | 16S-05 |  | C1-05 | ITS2-05 |
| 23 | *Dysmicoccus brevipes* | 1923 |  | MLH01 | 28S-01 | 16S-05 | LCO-20 | C1-05 | ITS2-05 |
| 23 | *Dysmicoccus brevipes* | 1924 |  | MLH01 | 28S-01 | 16S-05 | LCO-20 | C1-05 | ITS2-05 |
| 23 | *Dysmicoccus brevipes* | 1925 |  | MLH01 |  | 16S-05 | LCO-20 | C1-05 | ITS2-05 |
| 23 | *Dysmicoccus brevipes* | 1926 |  |  | 28S-01 |  |  | C1-05 |  |
| 23 | *Dysmicoccus brevipes* | 1927 |  | MLH01 | 28S-01 |  | LCO-20 | C1-05 |  |
| 38 | *Dysmicoccus brevipes* | 5008 | 1200840 | MLH01 | 28S-01 | 16S-05 | LCO-20 | C1-05 | ITS2-05 |
| 38 | *Dysmicoccus brevipes* | 5009 |  | MLH01 | 28S-01 | 16S-05 | LCO-20 | C1-05 | ITS2-05 |
| 38 | *Dysmicoccus brevipes* | 5010 |  | MLH01 | 28S-01 | 16S-05 | LCO-20 | C1-05 | ITS2-05 |
| 38 | *Dysmicoccus brevipes* | 5011 |  | MLH01 | 28S-01 | 16S-05 | LCO-20 | C1-05 | ITS2-05 |
| 38 | *Dysmicoccus brevipes* | 5012 |  | MLH01 | 28S-01 | 16S-05 | LCO-20 | C1-05 | ITS2-05 |
| 44 | *Dysmicoccus brevipes* | 5036 |  | MLH02 | 28S-01 |  |  | C1-06 |  |
| 44 | *Dysmicoccus brevipes* | 5037 | 1200841 | MLH02 | 28S-01 | 16S-05 | LCO-23 | C1-06 | ITS2-05 |
| 44 | *Dysmicoccus brevipes* | 5038 | 1200842 | MLH02 | 28S-01 | 16S-05 | LCO-23 | C1-06 | ITS2-05 |
| 44 | *Dysmicoccus brevipes* | 5039 | 1200843 | MLH02 | 28S-01 |  | LCO-23 | C1-06 | ITS2-05 |
| 44 | *Dysmicoccus brevipes* | 5040 |  | MLH02 | 28S-01 | 16S-05 | LCO-23 | C1-06 | ITS2-05 |
| 25 | *Dysmicoccus brevipes* | 5041 |  | MLH02 | 28S-01 |  |  | C1-06 |  |
| 25 | *Dysmicoccus brevipes* | 5042 |  |  | 28S-01 |  |  |  |  |
| 25 | *Dysmicoccus brevipes* | 5044 |  | MLH02 | 28S-01 |  |  | C1-06 |  |
| 10 | *Dysmicoccus sylvarum* | 1249 | 0902323 | MLH03 | 28S-17 | 16S-06 | LCO-26 | C1-19 | ITS2-01 |
| 10 | *Dysmicoccus sylvarum* | 1250 | 0902324 | MLH03 | 28S-17 | 16S-06 | LCO-26 | C1-19 | ITS2-01 |
| 10 | *Dysmicoccus sylvarum* | 1252 | 0902325 | MLH03 | 28S-17 | 16S-06 | LCO-26 | C1-19 | ITS2-01 |
| 11 | *Dysmicoccus sylvarum* | 1253 | 0902326 | MLH04 | 28S-17 | 16S-06 | LCO-27 | C1-20 | ITS2-01 |
| 11 | *Dysmicoccus sylvarum* | 1254 | 0902327 | MLH04 | 28S-17 | 16S-06 | LCO-27 | C1-20 | ITS2-01 |
| 11 | *Dysmicoccus sylvarum* | 1255 | 0902328 | MLH04 | 28S-17 | 16S-06 | LCO-27 | C1-20 | ITS2-01 |
| 11 | *Dysmicoccus sylvarum* | 1256 | 0902329 | MLH04 | 28S-17 | 16S-06 | LCO-27 | C1-20 | ITS2-01 |
| 11 | *Dysmicoccus sylvarum* | 1257 | 0902330 | MLH04 | 28S-17 | 16S-06 | LCO-27 | C1-20 | ITS2-01 |
| 4 | *Dysmicoccus texensis* | 1223 | 0902308 | MLH30 | 28S-15 | 16S-03 | LCO-17 | C1-18 | ITS2-02 |
| 33 | *Ferrisia meridionalis* | 1967 | 1200858 | MLH05 | 28S-03 | 16S-11 |  | C1-21 | ITS2-06 |
| 33 | *Ferrisia meridionalis* | 1968 | 1200859 | MLH05 | 28S-03 | 16S-11 |  | C1-21 | ITS2-06 |
| 33 | *Ferrisia meridionalis* | 1969 | 1200860 | MLH05 | 28S-03 | 16S-11 |  | C1-21 | ITS2-06 |
| 25 | *Ferrisia meridionalis* | 1976 | 1200861 | MLH05 | 28S-03 |  |  | C1-21 |  |
| 30 | *Ferrisia terani* | 1956 | 1200863 | MLH06 | 28S-04 | 16S-09 | LCO-22 | C1-22 | ITS2-07 |
| 33 | *Ferrisia terani* | 1970 | 1200864 | MLH07 | 28S-04 | 16S-09 | LCO-21 | C1-22 | ITS2-07 |
| 29 | *Ferrisia sp* | 1953 | 1200865 | MLH09 | 28S-05 |  |  | C1-12 | ITS2-03 |
| 29 | *Ferrisia sp* | 1955 | 1200866 | MLH09 | 28S-05 |  |  | C1-12 |  |
| 34 | *Ferrisia sp* | 1980 | 1200867 | MLH08 | 28S-05 | 16S-12 | LCO-14 | C1-13 | ITS2-04 |
| 33 | *Phenacoccus baccharidis* | 1971 | 1200888 | MLH33 | 28S-14 |  | LCO-28 | C1-26 | ITS2-19 |
| 37 | *Phenacoccus baccharidis* | 5007 | 1200889 | MLH34 | 28S-14 |  | LCO-25 | C1-27 | ITS2-18 |
| 24 | *Phenacoccus parvus* | 1928 | 1200885 | MLH10 | 28S-13 |  | LCO-24 | C1-24 | ITS2-11 |
| 24 | *Phenacoccus parvus* | 1930 |  | MLH10 | 28S-13 |  | LCO-24 | C1-24 | ITS2-11 |
| 24 | *Phenacoccus parvus* | 1931 |  | MLH10 | 28S-13 |  | LCO-24 | C1-24 | ITS2-11 |
| 24 | *Phenacoccus parvus* | 1932 |  | MLH10 | 28S-13 |  | LCO-24 | C1-24 | ITS2-11 |
| 22 | *Phenacoccus parvus* | 5003 |  | MLH10 | 28S-13 |  | LCO-24 | C1-24 | ITS2-11 |
| 22 | *Phenacoccus parvus* | 5004 | 1200886 | MLH10 | 28S-13 |  | LCO-24 | C1-24 | ITS2-11 |
| 22 | *Phenacoccus parvus* | 5005 |  | MLH10 | 28S-13 |  | LCO-24 | C1-24 | ITS2-11 |
| 22 | *Phenacoccus parvus* | 5006 |  | MLH10 | 28S-13 |  | LCO-24 | C1-24 | ITS2-11 |
| 46 | *Phenacoccus parvus* | 5051 | 1200887 | MLH10 | 28S-13 |  | LCO-24 | C1-24 | ITS2-11 |
| 25 | *Phenacoccus solenopsis* | 1973 | 1200879 | MLH11 | 28S-12 |  |  | C1-23 | ITS2-12 |
| 25 | *Phenacoccus solenopsis* | 1974 |  | MLH12 | 28S-12 |  |  | C1-23 | ITS2-13 |
| 25 | *Phenacoccus solenopsis* | 1975 |  | MLH12 | 28S-12 |  |  | C1-23 | ITS2-13 |
| 42 | *Phenacoccus solenopsis* | 5026 | 1200880 | MLH11 | 28S-12 |  |  | C1-23 | ITS2-12 |
| 42 | *Phenacoccus solenopsis* | 5027 | 1200881 | MLH12 | 28S-12 |  |  | C1-23 | ITS2-13 |
| 42 | *Phenacoccus solenopsis* | 5028 | 1200882 | MLH11 | 28S-12 |  |  | C1-23 | ITS2-12 |
| 42 | *Phenacoccus solenopsis* | 5029 |  |  | 28S-12 |  |  | C1-23 |  |
| 42 | *Phenacoccus solenopsis* | 5030 |  | MLH11 | 28S-12 |  |  | C1-23 | ITS2-12 |
| 45 | *Phenacoccus solenopsis* | 5046 |  |  | 28S-12 |  |  | C1-23 |  |
| 45 | *Phenacoccus solenopsis* | 5047 | 1200883 | MLH12 | 28S-12 |  |  | C1-23 | ITS2-13 |
| 45 | *Phenacoccus solenopsis* | 5048 | 1200884 | MLH12 | 28S-12 |  |  | C1-23 | ITS2-13 |
| 45 | *Phenacoccus solenopsis* | 5049 |  | MLH12 | 28S-12 |  |  | C1-23 | ITS2-13 |
| 45 | *Phenacoccus solenopsis* | 5050 |  | MLH12 | 28S-12 |  |  | C1-23 | ITS2-13 |
| 19 | *Planococcus citri* | 1714 | 1101834 | MLH13 | 28S-02 | 16S-07 | LCO-01 | C1-03 | ITS2-14 |
| 19 | *Planococcus citri* | 1715 |  | MLH13 |  | 16S-07 | LCO-01 | C1-03 | ITS2-14 |
| 19 | *Planococcus citri* | 1716 | 1101836 | MLH13 | 28S-02 | 16S-07 | LCO-01 | C1-03 | ITS2-14 |
| 19 | *Planococcus citri* | 1717 | 1101837 | MLH13 | 28S-02 | 16S-07 | LCO-01 | C1-03 | ITS2-14 |
| 20 | *Planococcus citri* | 1817 |  | MLH13 | 28S-02 | 16S-07 | LCO-01 | C1-03 | ITS2-14 |
| 21 | *Planococcus citri* | 1819 |  | MLH13 | 28S-02 | 16S-07 | LCO-01 | C1-03 | ITS2-14 |
| 21 | *Planococcus citri* | 1820 |  | MLH13 | 28S-02 | 16S-07 | LCO-01 | C1-03 | ITS2-14 |
| 21 | *Planococcus citri* | 1821 |  | MLH13 | 28S-02 | 16S-07 | LCO-01 | C1-03 | ITS2-14 |
| 21 | *Planococcus citri* | 1822 |  | MLH13 | 28S-02 | 16S-07 | LCO-01 | C1-03 | ITS2-14 |
| 22 | *Planococcus citri* | 1823 |  | MLH13 | 28S-02 | 16S-07 | LCO-01 | C1-03 | ITS2-14 |
| 22 | *Planococcus citri* | 1824 |  | MLH13 |  |  |  | C1-03 |  |
| 22 | *Planococcus citri* | 1825 |  | MLH13 | 28S-02 |  | LCO-01 | C1-03 | ITS2-14 |
| 22 | *Planococcus citri* | 1826 |  | MLH13 | 28S-02 |  |  | C1-03 | ITS2-14 |
| 25 | *Planococcus citri* | 1933 |  | MLH13 | 28S-02 |  |  | C1-03 |  |
| 26 | *Planococcus citri* | 1936 | 1200844 | MLH16 | 28S-02 |  | LCO-04 | C1-01 |  |
| 26 | *Planococcus citri* | 1937 |  | MLH16 | 28S-02 |  | LCO-04 | C1-01 | ITS2-14 |
| 26 | *Planococcus citri* | 1938 |  | MLH16 |  | 16S-07 | LCO-04 | C1-01 | ITS2-14 |
| 26 | *Planococcus citri* | 1939 | 1200845 |  | 28S-02 | 16S-07 |  | C1-01 | ITS2-14 |
| 26 | *Planococcus citri* | 1940 | 1200846 | MLH16 | 28S-02 | 16S-07 | LCO-04 | C1-01 | ITS2-14 |
| 27 | *Planococcus citri* | 1941 |  |  |  |  | LCO-04 |  | ITS2-14 |
| 27 | *Planococcus citri* | 1942 | 1200847 | MLH18 | 28S-02 |  | LCO-05 | C1-01 | ITS2-14 |
| 27 | *Planococcus citri* | 1943 | 1200894 | MLH18 |  | 16S-07 | LCO-05 |  | ITS2-14 |
| 27 | *Planococcus citri* | 1944 |  | MLH18 | 28S-02 | 16S-07 | LCO-05 | C1-01 | ITS2-14 |
| 27 | *Planococcus citri* | 1945 |  |  | 28S-02 | 16S-07 |  | C1-01 | ITS2-14 |
| 28 | *Planococcus citri* | 1946 |  | MLH13 | 28S-02 | 16S-07 | LCO-01 | C1-03 | ITS2-14 |
| 28 | *Planococcus citri* | 1947 |  | MLH13 | 28S-02 | 16S-07 | LCO-01 | C1-03 | ITS2-14 |
| 28 | *Planococcus citri* | 1948 |  | MLH13 | 28S-02 | 16S-07 | LCO-01 | C1-03 | ITS2-14 |
| 28 | *Planococcus citri* | 1949 |  | MLH13 | 28S-02 |  |  | C1-03 | ITS2-14 |
| 28 | *Planococcus citri* | 1950 |  | MLH13 | 28S-02 | 16S-07 | LCO-01 | C1-03 | ITS2-14 |
| 29 | *Planococcus citri* | 1951 | 1200848 | MLH14 | 28S-02 | 16S-07 | LCO-02 | C1-04 | ITS2-14 |
| 29 | *Planococcus citri* | 1954 | 1200849 | MLH17 | 28S-02 | 16S-07 | LCO-04 | C1-02 | ITS2-14 |
| 32 | *Planococcus citri* | 1962 | 1200850 | MLH16 | 28S-02 | 16S-07 | LCO-04 | C1-01 | ITS2-14 |
| 34 | *Planococcus citri* | 1977 |  |  | 28S-02 | 16S-07 |  | C1-01 | ITS2-14 |
| 34 | *Planococcus citri* | 1978 | 1200851 | MLH16 | 28S-02 | 16S-07 | LCO-04 | C1-01 | ITS2-14 |
| 34 | *Planococcus citri* | 1979 |  | MLH16 | 28S-02 | 16S-07 | LCO-04 | C1-01 | ITS2-14 |
| 34 | *Planococcus citri* | 1981 |  |  | 28S-02 |  |  | C1-01 |  |
| 25 | *Planococcus citri* | 1983 |  | MLH13 | 28S-02 |  | LCO-01 | C1-03 |  |
| 25 | *Planococcus citri* | 1986 |  | MLH13 | 28S-02 |  |  | C1-03 |  |
| 35 | *Planococcus citri* | 1988 |  | MLH13 | 28S-02 | 16S-07 | LCO-01 | C1-03 | ITS2-14 |
| 35 | *Planococcus citri* | 1989 |  | MLH13 | 28S-02 | 16S-07 | LCO-01 | C1-03 | ITS2-14 |
| 35 | *Planococcus citri* | 1990 |  | MLH13 | 28S-02 | 16S-07 | LCO-01 | C1-03 | ITS2-14 |
| 35 | *Planococcus citri* | 1991 |  | MLH13 | 28S-02 | 16S-07 | LCO-01 | C1-03 | ITS2-14 |
| 35 | *Planococcus citri* | 1992 |  | MLH13 | 28S-02 |  | LCO-01 | C1-03 | ITS2-14 |
| 36 | *Planococcus citri* | 1993 |  | MLH13 | 28S-02 | 16S-07 | LCO-01 | C1-03 | ITS2-14 |
| 36 | *Planococcus citri* | 1994 |  | MLH13 | 28S-02 | 16S-07 | LCO-01 | C1-03 | ITS2-14 |
| 36 | *Planococcus citri* | 1995 |  | MLH13 | 28S-02 | 16S-07 | LCO-01 | C1-03 | ITS2-14 |
| 36 | *Planococcus citri* | 1996 |  | MLH13 | 28S-02 | 16S-07 | LCO-01 | C1-03 | ITS2-14 |
| 36 | *Planococcus citri* | 1997 |  | MLH13 | 28S-02 | 16S-07 | LCO-01 | C1-03 | ITS2-14 |
| 25 | *Planococcus citri* | 1999 |  | MLH13 | 28S-02 |  |  | C1-03 |  |
| 25 | *Planococcus citri* | 2000 |  | MLH13 |  |  |  | C1-03 |  |
| 25 | *Planococcus citri* | 5001 |  | MLH13 |  |  |  | C1-03 |  |
| 25 | *Planococcus citri* | 5002 |  | MLH13 |  |  |  | C1-03 |  |
| 40 | *Planococcus citri* | 5016 | 1200852 | MLH15 | 28S-02 | 16S-07 | LCO-03 | C1-01 | ITS2-14 |
| 40 | *Planococcus citri* | 5017 | 1200853 | MLH15 | 28S-02 | 16S-07 | LCO-03 | C1-01 | ITS2-14 |
| 40 | *Planococcus citri* | 5018 | 1200854 | MLH15 | 28S-02 | 16S-07 | LCO-03 | C1-01 | ITS2-14 |
| 40 | *Planococcus citri* | 5019 |  | MLH15 | 28S-02 | 16S-07 | LCO-03 | C1-01 | ITS2-14 |
| 40 | *Planococcus citri* | 5020 |  | MLH13 | 28S-02 |  | LCO-01 | C1-03 |  |
| 41 | *Planococcus citri* | 5021 | 1200855 | MLH14 | 28S-02 | 16S-07 | LCO-02 | C1-04 | ITS2-14 |
| 41 | *Planococcus citri* | 5022 | 1200856 | MLH14 | 28S-02 | 16S-07 | LCO-02 | C1-04 | ITS2-14 |
| 41 | *Planococcus citri* | 5023 |  | MLH14 | 28S-02 | 16S-07 | LCO-02 | C1-04 | ITS2-14 |
| 41 | *Planococcus citri* | 5024 | 1200857 | MLH14 | 28S-02 | 16S-07 | LCO-02 | C1-04 | ITS2-14 |
| 48 | *Planococcus citri* | 5055 |  | MLH14 |  |  | LCO-02 | C1-04 | ITS2-14 |
| 48 | *Planococcus citri* | 5056 |  | MLH14 | 28S-02 | 16S-07 | LCO-02 | C1-04 | ITS2-14 |
| 48 | *Planococcus citri* | 5057 |  | MLH14 | 28S-02 | 16S-07 | LCO-02 | C1-04 | ITS2-14 |
| 48 | *Planococcus citri* | 5058 |  | MLH14 |  |  | LCO-02 | C1-04 |  |
| 32 | *Pseudococcus cryptus* | 1963 | 1200874 | MLH19 | 28S-09 | 16S-10 | LCO-19 | C1-14 |  |
| 32 | *Pseudococcus cryptus* | 1964 | 1200875 | MLH19 | 28S-09 | 16S-10 |  | C1-14 |  |
| 32 | *Pseudococcus cryptus* | 1965 | 1200876 | MLH19 | 28S-09 | 16S-10 |  | C1-14 |  |
| 32 | *Pseudococcus cryptus* | 1966 | 1200877 | MLH19 | 28S-09 | 16S-10 |  | C1-14 |  |
| 3 | *Pseudococcus* nr. *maritimus* | 1222 | 0902307 | MLH35 | 28S-16 | 16S-02 | LCO-18 | C1-17 | ITS2-10 |
| 43 | *Pseudococcus* nr. *meridionalis* | 5031 | 1200890 | MLH31 | 28S-19 |  |  |  | ITS2-08 |
| 43 | *Pseudococcus* nr. *meridionalis* | 5032 | 1200891 | MLH31 | 28S-19 |  |  | C1-25 | ITS2-08 |
| 43 | *Pseudococcus* nr. *meridionalis* | 5033 | 1200892 | MLH31 | 28S-19 |  |  | C1-25 | ITS2-08 |
| 43 | *Pseudococcus* nr. *meridionalis* | 5034 | 1200893 | MLH31 | 28S-19 |  |  | C1-25 | ITS2-08 |
| 43 | *Pseudococcus* nr. *meridionalis* | 5035 |  | MLH31 |  |  |  | C1-25 | ITS2-08 |
| 20 | *Pseudococcus* nr. *sociabilis* | 1815 | 1101838 | MLH29 | 28S-11 |  | LCO-13 | C1-15 |  |
| 20 | *Pseudococcus* nr. *sociabilis* | 1816 | 1101839 | MLH28 | 28S-10 |  |  | C1-16 | ITS2-09 |
| 29 | *Pseudococcus* nr. *sociabilis* | 1952 | 1200878 | MLH28 | 28S-10 | 16S-08 |  | C1-16 | ITS2-09 |
| 6 | *Pseudococcus* nr. *viburni* | 1230 | 0902313 | MLH27 | 28S-07 | 16S-04 |  | C1-28 | ITS2-17 |
| 6 | *Pseudococcus* nr. *viburni* | 1232 | 0902315 | MLH27 | 28S-07 | 16S-04 | LCO-15 |  | ITS2-17 |
| 16 | *Pseudococcus sp* | 1710 | 1101830 | MLH32 | 28S-18 |  | LCO-16 | C1-29 | ITS2-20 |
| 1 | *Pseudococcus viburni* | 1215 | 0902302 | MLH26 | 28S-08 | 16S-01 | LCO-06 | C1-07 | ITS2-15 |
| 2 | *Pseudococcus viburni* | 1217 |  | MLH26 | 28S-08 | 16S-01 | LCO-06 | C1-07 | ITS2-15 |
| 2 | *Pseudococcus viburni* | 1218 | 0902303 | MLH26 | 28S-08 | 16S-01 | LCO-06 | C1-07 | ITS2-15 |
| 2 | *Pseudococcus viburni* | 1219 | 0902305 | MLH26 | 28S-08 | 16S-01 | LCO-06 | C1-07 | ITS2-15 |
| 2 | *Pseudococcus viburni* | 1220 | 0902306 | MLH26 | 28S-08 | 16S-01 |  | C1-07 | ITS2-15 |
| 2 | *Pseudococcus viburni* | 1221 |  | MLH26 | 28S-08 | 16S-01 | LCO-06 | C1-07 | ITS2-15 |
| 5 | *Pseudococcus viburni* | 1224 | 0902309 | MLH24 | 28S-06 | 16S-01 | LCO-11 | C1-09 | ITS2-16 |
| 5 | *Pseudococcus viburni* | 1225 | 1200908 | MLH24 | 28S-06 | 16S-01 | LCO-11 |  | ITS2-16 |
| 5 | *Pseudococcus viburni* | 1227 | 0902310 | MLH24 | 28S-06 | 16S-01 | LCO-11 | C1-09 | ITS2-16 |
| 5 | *Pseudococcus viburni* | 1228 | 0902311 | MLH24 | 28S-06 | 16S-01 | LCO-11 | C1-09 | ITS2-16 |
| 5 | *Pseudococcus viburni* | 1229 | 0902312 |  | 28S-06 | 16S-01 |  | C1-09 | ITS2-16 |
| 6 | *Pseudococcus viburni* | 1231 | 0902314 | MLH26 | 28S-08 | 16S-01 | LCO-06 | C1-07 | ITS2-15 |
| 12 | *Pseudococcus viburni* | 1258 | 0902331 | MLH20 | 28S-06 | 16S-01 | LCO-07 | C1-11 | ITS2-16 |
| 12 | *Pseudococcus viburni* | 1259 | 1101681 | MLH20 | 28S-06 | 16S-01 | LCO-07 | C1-11 |  |
| 13 | *Pseudococcus viburni* | 1260 | 0902332 | MLH23 | 28S-06 | 16S-01 | LCO-10 | C1-09 | ITS2-16 |
| 13 | *Pseudococcus viburni* | 1262 | 1101682 | MLH21 | 28S-06 | 16S-01 | LCO-08 | C1-10 | ITS2-16 |
| 13 | *Pseudococcus viburni* | 1263 | 0902334 |  | 28S-06 | 16S-01 |  | C1-09 |  |
| 13 | *Pseudococcus viburni* | 1264 |  |  | 28S-06 |  |  | C1-09 | ITS2-16 |
| 15 | *Pseudococcus viburni* | 1708 |  | MLH26 | 28S-08 |  | LCO-06 |  | ITS2-15 |
| 15 | *Pseudococcus viburni* | 1709 |  | MLH26 |  |  | LCO-06 |  | ITS2-15 |
| 17 | *Pseudococcus viburni* | 1711 |  | MLH26 | 28S-08 | 16S-01 | LCO-06 | C1-07 | ITS2-15 |
| 18 | *Pseudococcus viburni* | 1712 | 1101832 | MLH25 | 28S-06 |  | LCO-12 |  | ITS2-16 |
| 18 | *Pseudococcus viburni* | 1713 |  | MLH26 |  |  |  | C1-07 |  |
| 31 | *Pseudococcus viburni* | 1957 |  | MLH26 | 28S-08 |  |  | C1-07 | ITS2-15 |
| 31 | *Pseudococcus viburni* | 1958 |  | MLH26 | 28S-08 | 16S-01 | LCO-06 | C1-07 | ITS2-15 |
| 31 | *Pseudococcus viburni* | 1959 |  | MLH26 | 28S-08 |  |  | C1-07 | ITS2-15 |
| 31 | *Pseudococcus viburni* | 1960 | 1200862 | MLH26 | 28S-08 | 16S-01 |  | C1-07 | ITS2-15 |
| 31 | *Pseudococcus viburni* | 1961 | 1200873 | MLH26 | 28S-08 | 16S-01 | LCO-06 | C1-07 | ITS2-15 |
| 39 | *Pseudococcus viburni* | 5013 |  | MLH26 | 28S-08 | 16S-01 | LCO-06 | C1-07 | ITS2-15 |
| 39 | *Pseudococcus viburni* | 5014 |  | MLH26 | 28S-08 | 16S-01 | LCO-06 | C1-07 | ITS2-15 |
| 39 | *Pseudococcus viburni* | 5015 |  | MLH26 | 28S-08 | 16S-01 | LCO-06 | C1-07 | ITS2-15 |
| 47 | *Pseudococcus viburni* | 5052 | 1200870 | MLH24 | 28S-06 |  | LCO-11 |  | ITS2-16 |
| 47 | *Pseudococcus viburni* | 5053 | 1200871 | MLH22 | 28S-06 |  | LCO-09 | C1-08 |  |
| 47 | *Pseudococcus viburni* | 5054 |  | MLH22 |  |  | LCO-09 |  |  |


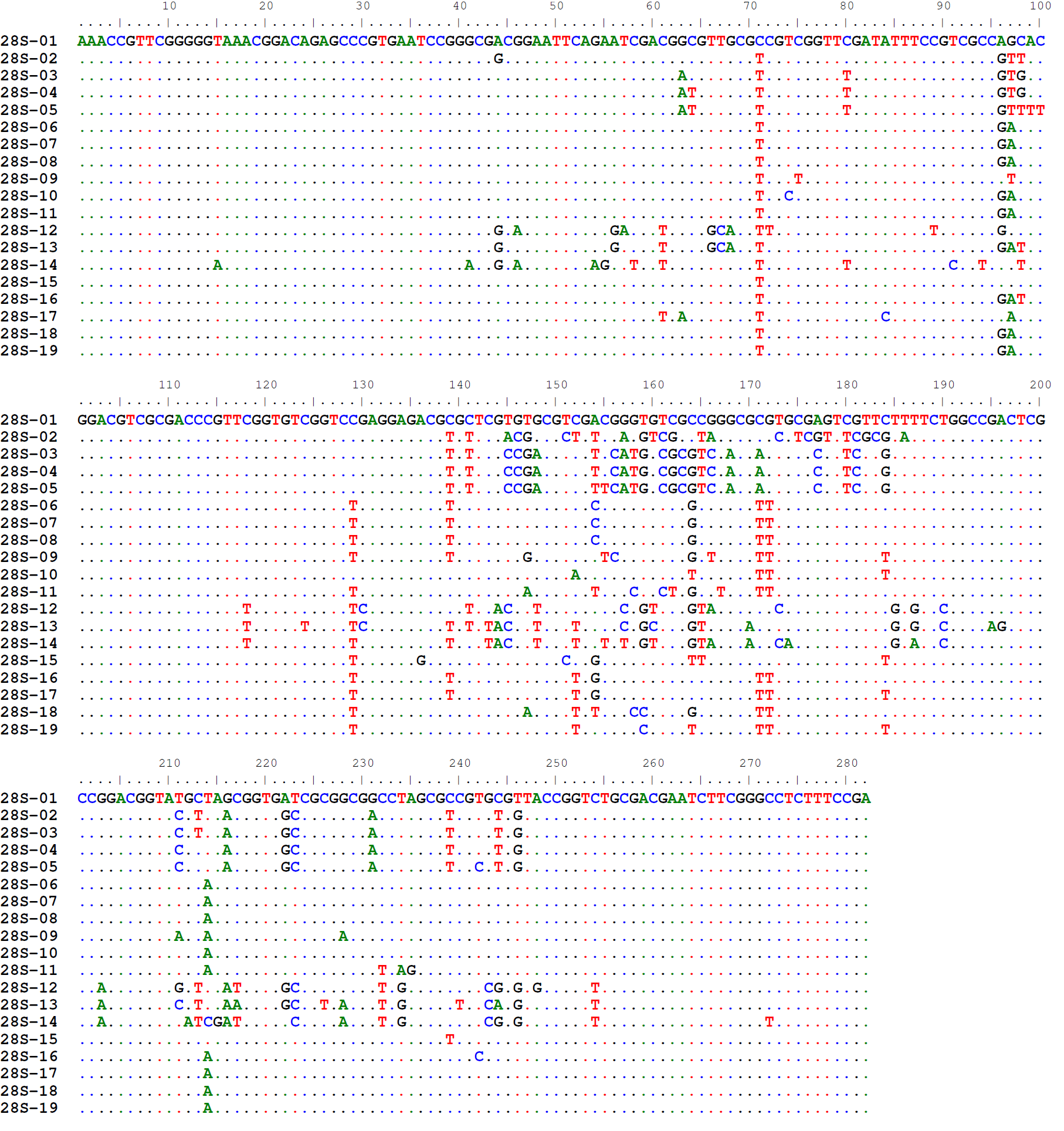


Figure S1. 28S sequence alignment used to calculate the Neighbor joining tree of Figure 1. Regions of the alignment with insertions / deletions are removed.
